# Molecular surveillance of antimicrobial resistance and transmission pattern of *Mycobacterium leprae* in Chinese leprosy patients

**DOI:** 10.1080/22221751.2019.1677177

**Published:** 2019-10-17

**Authors:** Santosh Chokkakula, Zhiming Chen, Le Wang, Haiqin Jiang, Yanqing Chen, Ying Shi, Wenyue Zhang, Wei Gao, Jun Yang, Jinlan Li, Xiong Li, Tiejun Shui, Jun He, Limei Shen, Jie Liu, De Wang, Hao Wang, Huan Chen, Yanfei Kuang, Bin Li, Ziyi Chen, Aiping Wu, Meiwen Yu, Liangbin Yan, Naveen Chandra Suryadevara, Varalakshmi Vissa, Weida Liu, Hongsheng Wang

**Affiliations:** a Institute of Dermatology, Chinese Academy of Medical Sciences and Peking Union Medical College, Nanjing, China; b National Centre for STD and Leprosy Control, China CDC, Nanjing, China; c Jiangsu Key Laboratory of Molecular Biology for Skin Diseases and STIs, Nanjing, China; d Yunnan Provincial CDC, Kunming, China; e Guizhou Provincial CDC, Guiyang, China; f Sichuan Provincial People’s Hospital, Chengdu, China; g Hunan Provincial CDC, Changsha, China; h Suzhou Institute of Systems Medicine, Suzhou, China; i Vanderbilt University Medical Centre, Nashville, TN, USA; j Centre for global health, School of Public Health, Nanjing Medical University, Nanjing, China

**Keywords:** *M. leprae*, antimicrobial resistance, bacteriological index, genotyping, transmission

## Abstract

Reports on antimicrobial resistance (AMR) of *Mycobacterium leprae,* relationship with bacteriological index (BI), and transmission in China are limited. We investigated the emergence of AMR mutations, the relationship between BI and AMR in complete, moderate and lack of BI decline cases, and molecular epidemiological features of AMR cases by enrolling 290 leprosy cases from four endemic provinces. Seven (2.41%), one (0.34%), five (1.72%), one (0.34%), and one (0.34%) strains had single mutations in *folP1*, *rpoC*, *gyrA*, *gyrB*, and *23S rRNA,* respectively. Double mutations in *folP1* and *gyrA*, *rpoB* and *gyrA*, and *gyrA* and *23S rRNA* were observed in one (0.34%) strain each. Mutated strains occurred in three out of 81 (95% CI−0.005-0.079, *p* = 0.083) cases with complete BI decline, in seven out of 103 (95% CI 0.018-0.117, *p* = 0.008) cases with moderate BI decline, and in four out of 34 (95% CI 0.003-0.231, *p* = 0.044) cases with lack of BI decline. Most of these mutated strains were geographically separated and diverged genotypically. AMR mutations may not be the main cause of the lack of BI decline. The low transmission of AMR strains at the county level indicates an ongoing transmission at close contact levels.

## Introduction

Leprosy is a chronic granulomatous disease that mainly affects the skin, peripheral nerves, and mucous membrane [1]. The introduction of multidrug therapy (MDT) with dapsone, rifampicin, and clofazimine has decreased the prevalence of leprosy worldwide. However, more than 216,108 new leprosy cases were reported in 2016, indicating the continuous transmission of the disease [2]. According to National reports, China estimated 634 new cases in 2017 [2]. The reduction in leprosy prevalence has been observed in recent years in China; however, a considerable number of new, relapse, and drug-resistant cases occurred representing a major public health concern [3].

Drug resistance detection of WHO-recommended DRDRs containing *folP1*, *rpoB*, and *gyrA* associated with dapsone, rifampicin, and ofloxacin resistance respectively is well established and has been incorporated in the WHO guidelines for AMR surveillance. There are several AMR reports available regarding dapsone [4], rifampicin [5], and ofloxacin [6] resistance in leprosy. In addition to mutations in WHO-recommended DRDRs, compensatory AMR-associated mutations, including *nth* (DNA repair), *rpoA* (rifampicin), *rpoB, rpoC* (rifampicin), *gyrA, gyrB* (ofloxacin), and *23S rRNA* (clarithromycin) have been reported in *Mycobacterium tuberculosis* and *Mycobacterium leprae*. However, the roles of these mutations in *M. leprae* are yet to be confirmed [7–9].

The BI measurement in the slit-skin smear of patients with potential leprosy is used for diagnosis, classification, and clinical recovery and relapse detection. BI at diagnosis and during treatment, release from treatment (RFT), and follow-up have previously been studied to justify the efficacy of MDT. Generally, 6-months and 1-year MDT courses are recommended for paucibacillary (PB) and multibacillary (MB) cases respectively. Nevertheless, no effective BI change was observed in some leprosy cases, even if MDT was prescribed for more than 1-year (Supplementary Table 1) [10–19]. The factors influencing the lack of BI decline remain unknown, but high initial BI and no decline in BI at RFT are possible signs of AMR [12].

Molecular epidemiological studies for strain typing of *M. leprae* have been well developed to track strain transmission and geographical distribution in endemic and non-endemic regions [20]. *M. leprae* isolates having identical genotypes are expected to be associated with recent transmission. To our knowledge, no studies on the transmission of AMR strains of *M. leprae* at the county level in China have been performed.

In the current study, we analysed the AMR of *M. leprae* by direct PCR sequencing of the WHO-recommended DRDRs containing *folP1*, *rpoB*, and *gyrA*. We also investigated the sequence of regions and genes of extended DRDRs including *nth, rpoA*, *rpoB*, *rpoC*, *gyrA, gyrB*, and *23S rRNA* to evaluate the necessity to complement the current surveillance system for AMR mutations. AMR mutations were further studied for their relationship with BI and to identify mutation trends in new and relapse cases. Finally, we performed a population-based study of leprosy by using genomic VNTR and epidemiological features of wild-type and mutated strains of *M. leprae* at the county level to examine the transmission of mutated strains of leprosy in China.

## Materials and methods

### Study sites

**Figure 1.**
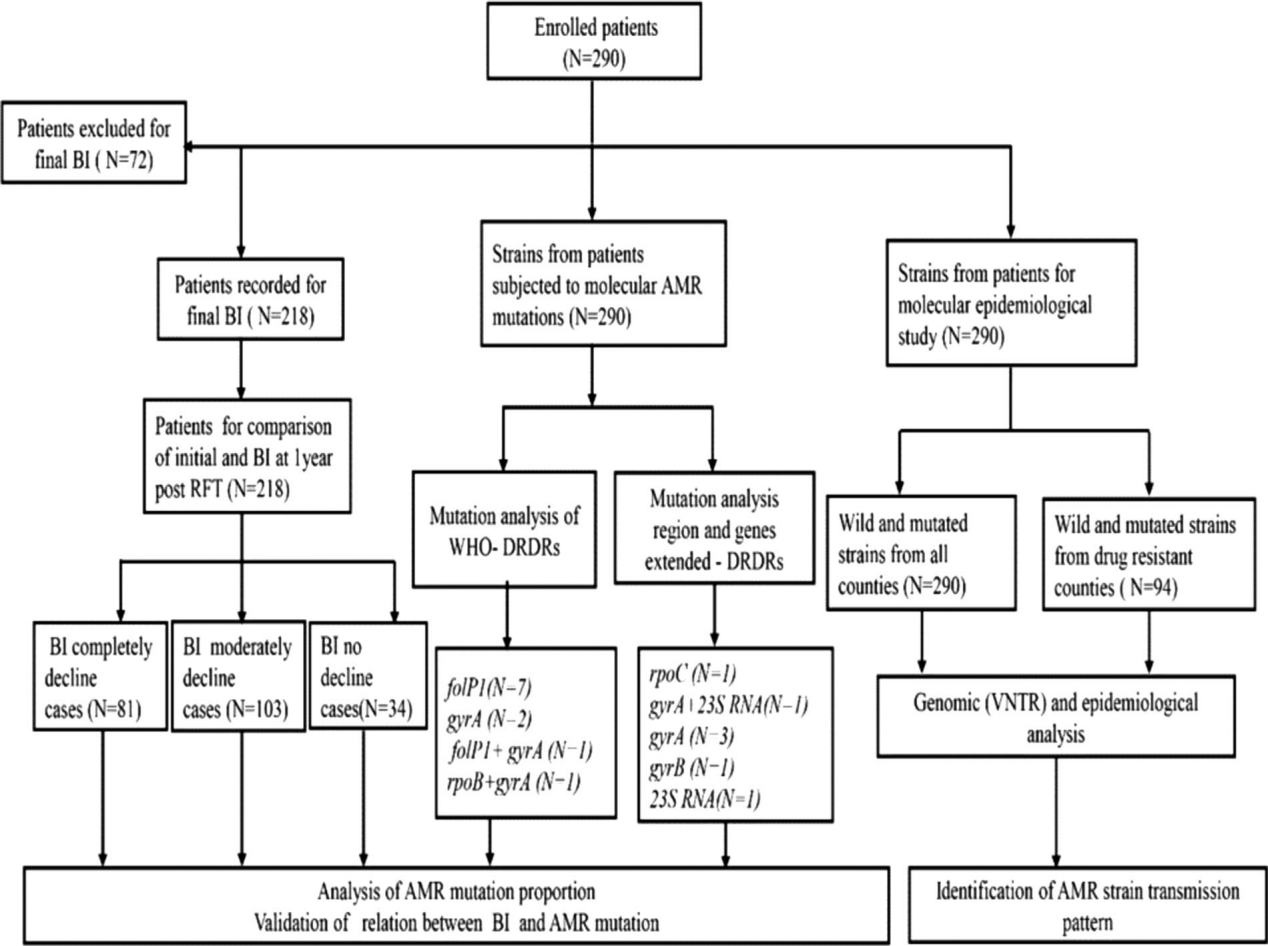
Schematic procedure and structure of the study. BI status at diagnosis was compared to BI at 1-year post-RFT, which identified three categorized patients: complete decline, moderate decline, and lack of BI decline. Patients under treatment were excluded from this grouping. *M. leprae* VNTR typing and molecular drug resistance analysis were performed for all 290 patients.

**Figure 2.**
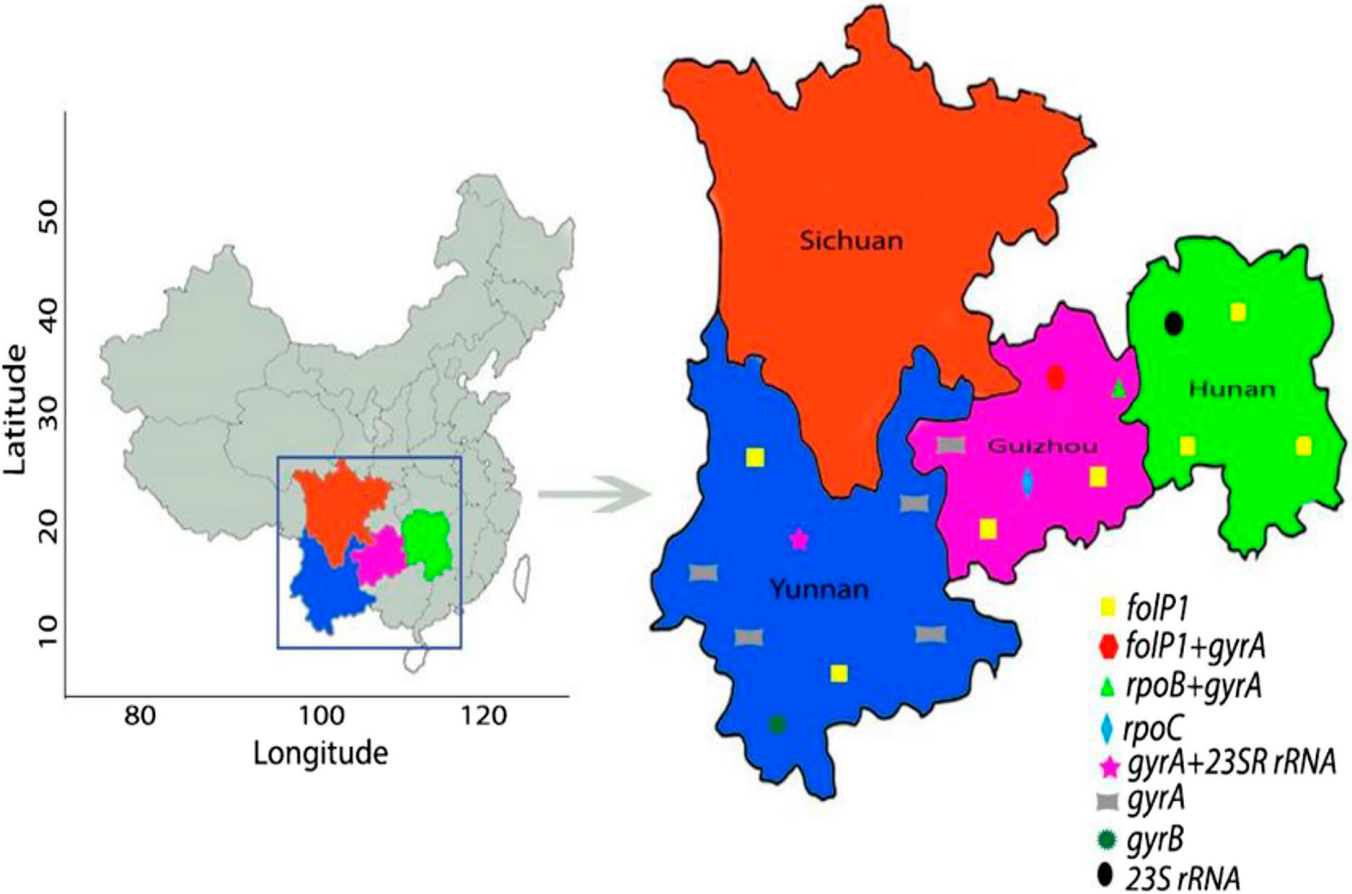
Study sites and geographical distribution of AMR strains of *M. leprae*. Four provinces shown in colour were included in the surveillance for AMR of leprosy. AMR mutations were not detected in Sichuan province. All AMR mutation types detected in Yunnan, Guizhou, and Hunan provinces are indicated.

The detailed study profile is depicted in Figure 1. The study was conducted under the national AMR surveillance programme from 2013 to 2017 in four endemic provinces namely Guizhou, Hunan, Sichuan, and Yunnan. These provinces account for > 80% of all leprosy cases in China. Some of the districts of these provinces have a high burden of leprosy with a prevalence rate is about > 1/100,000. The study area is located between the 20° N and 30° N latitudes and has tropical and subtropical climate conditions (Figure 2).

### Patient enrolment, BI monitoring, and follow-up of cases

During the study period, 270 newly diagnosed and 20 relapse cases of leprosy in four provinces were enrolled. For relapse screening, retrospective data of RFT patients from 2005 to 2010 were collected. Diagnosis and prescription of PB and MB-MDT regimen were performed as per WHO guidelines. Initial BI was recorded at the time of MDT regimen initiation, and cases were followed up by professional health workers periodically until the completion of 6-months or 1-year MDT. The BI was assessed at the time of RFT and 1-year post-RFT. Three categories namely complete BI decline, moderate BI decline, and lack of BI decline were categorized according to BI comparison at initial and 1-year post-RFT.

### PCR and sequencing of DRDRs

The presence of *M. leprae* genomic DNA was confirmed by PCR detection of the *16S rRNA* gene [21]. PCR and nested PCR were performed to amplify the WHO-recommended DRDRs (*folP1*, *rpoB*, and *gyrA*) and regions and genes of extended DRDRs including *nth*, *rpoA*, *rpoB*, *rpoC*, *gyrA*, *gyrB*, and *23S rRNA* by using primers and conditions listed in Supplementary Tables 2–6. The quality of amplified products was assessed using 1–2% agarose gel. PCR amplicons were purified and sequenced at the local commercial sequencing company (Tsingke Biological Technology Co., Ltd., Nanjing, China). Alignments to NCBI nucleotide databases were performed using the basic local alignment sequence tool (NCBI-BLAST) to detect the mutations.

### Molecular epidemiological analysis

The strain types and geographical distances within and between counties and provinces were analysed. Counties with drug-resistant strains were further studied and correlated with other counties, forming clear clusters with drug-resistant strains. Seventeen VNTR loci, AC8b, (GTA)9, (GGT)5, (AT)17, *rpoT*, 21-3, (AC)9, (AT)15, (AC)8a, 27-5, 6-7, (TA)18, (TTC)21,18-8, 12-5, 23-3, and (TA)10 were used to perform genotyping of *M. leprae* strains [22]. A phylogenetic tree was constructed using the ggtree package of R using the neighbour joining method.

### Statistical analysis

Data were statistically analysed using IBM SPSS software version 22. The Chi-square test was used to compare the parametric continuous data amongst different study groups. *P* < 0.05 was considered statistically significant.

## Results

### Clinical and BI details of MDT completed cases and cases under treatment

The demographical and clinical details of the patients from the four provinces are shown in Table 1. Amongst the 290 cases, 218 had completed MDT, and the rest were under treatment. Comparison of initial BI to BI at RFT for each of the 218 patients indicated three trends: complete decline, moderate decline, and lack of decline (including increase). Initial BIs (measured on a scale of 0–6) were stratified by one unit intervals, and their levels at RFT and follow-up are plotted in Supplementary Figure 1. Most of the BI distributions showed a gradual BI decline pattern from initial to 1-year post-RFT, except 5 to < 6 BI distribution, exhibiting a rapid BI decline pattern from initial to 1-year post-RFT. On the other hand, we observed a gradual BI decline pattern from RFT to 1-year post-RFT in all BI distributions, including 5 to < 6. The mean initial and RFT BIs of 81 cases (including 38 cases with initial BI zero), whose complete BI declines were 1.08 (range 0–4.8) and 0.21 (range 0–3.6) respectively were found to be BI negative and non-symptomatic at 1-year post-RFT. In 103 cases with moderate BI decline, the mean initial BI was 3.82 (range 1.2–5.7), which declined to 2.5 (range 0.4–4.8) at RFT and further to 2.04 (range 0.2–4.3) at 1-year post-RFT. A total of 34 cases (including 23 cases with increased BI) had BIs that did not decline, and they did not exhibit any clinical improvement. The mean BI at initial, RFT, and 1-year post-RFT were 2.65 (range 0–5.8), 3.45 (range 1–5.8), and 3.45 (range 1–5.8) respectively (Table 2). A different plot shows BI distribution at three-time points for complete, moderate, and lack of BI decline (Supplementary Figure 2). In the complete BI decline group, BI rapidly declined from the initial to the RFT and remained stable towards the 1-year post-RFT. The stable BI decline pattern at three time periods, including initial, RFT, and 1-year post-RFT were observed in the moderately BI decline group. Instead of stable BI pattern from initial to RFT in the lack of BI decline group, we observed a rise in BI from initial to RFT due to increased BI load during the treatment in some cases of lack of BI decline (Supplementary Figure 2). Other cases are currently under treatment, and their relevant details are summarized in Supplementary Table 6.

**Table 1.** Clinical and other relative information of all cases from four provinces.

Clinical characteristics	Hunan N (%)	Sichuan N (%)	Guizhou N (%)	Yunnan N (%)	Total N (%)
*Case type*					
New	28 (9.7)	21 (7.2)	116 (40)	105 (36.2)	270 (93.1)
Relapse	3 (1.03)	0 (0)	10 (3.5)	7 (2.41)	20 (6.9)
*Gender*					
Male	19 (6.6)	15 (5.1)	89 (30.7)	77 (26.6)	200 (69)
Female	12 (4.1)	6 (2.1)	37 (12.8)	35 (12)	90 (31)
Age (mean)	47	47	37	39	42.5
*R -J classification*					
TT	1 (0.3)	2 (0.7)	3 (1)	7 (2.4)	13 (4.4)
BT	2 (0.7)	4 (1.4)	16 (5.6)	31 (10.7)	53 (18.4)
BB	1 (0.3)	1 (0.3)	8 (2.8)	6 (2)	16 (5.4)
BL	12 (4.1)	7 (2.4)	60 (20.7)	57 (19.7)	136 (46.9)
LL	15 (5.2)	7 (2.4)	39 (13.5)	11 (3.8)	72(24.9)
*Nerve involvement*					
Yes	22 (7.6)	15 (5.1)	87 (30)	96 (33.1)	220 (75.8)
No	9 (3.1)	6 (2)	39 (13.5)	16 (5.6)	70 (24.2)
*Deformity*					
Yes	13 (4.4)	8 (2.8)	49 (16.9)	33 (11.4)	103 (35.5)
No	18 (6.2)	13 (4.4)	77 (26.6)	79 (27.3)	187 (64.5)
*Reaction*					
Yes	9 (3.1)	2 (0.8)	12 (4.1)	17 (5.8)	40 (13.8)
No	22 (7.6)	19 (6.6)	114 (39.3)	95 (32.7)	250 (86.2)

Number (percentage); TT-tuberculoid tuberculoid leprosy, BT-borderline tuberculoid leprosy, BB-borderline borderline leprosy, BL-borderline lepromatous leprosy, LL-lepromatous leprosy.

**Table 2.** Clinical characteristics and AMR results of BI completely decline, BI moderately decline, and lack of BI decline cases of leprosy.

Clinical characteristics	BI completely declines N (%)	BI moderately decline N (%)	Lack of BI decline N (%)	*p*-value
*Gender*				*p* = 0.833
Male	57 (26.1)	75 (34.5)	23 (10.6)	
Female	24 (11)	28 (12.8)	11 (5)	
*WHO Classification*				
MB	66 (30.2)	103 (47.3)	33 (15.1)	*p* = 0.000
PB	15 (6.9)	0 (0)	1 (0.5)	
Initial BI*	1.08 (0–4.8.0)	3.82(1.2.–5.7)	2.65 (0–5.8.0)	*p* = 0.000
BI at RFT*	0.21 (0–3.6)	2.50 (0.4–4.8)	3.45 (1–5.8.0)	*p* = 0.855
BI at 1 year post RFT*	0 (0)	2.04 (0.2–4.3)	3.45 (1–5.8.0)	*p* = 0.000
*16S rRNA PCR*				
Positive	81 (100)	103 (100)	34 (100)	*p* = 0.000
Negative	0	0	0	
*AMR mutations*				
*folP1*	1 (0.5)	5 (2.2)	0	*p* = 0.268
*folP1+ gyrA*	0 (0)	0 (0)	0	
*rpoB *+ *gyrA*	0 (0)	1 (0.5)	0	
*rpoC*	1 (0.5)	0 (0)	0	
*gyrA + 23S rRNA*	0	0 (0)	0	
*gyrA*	1 (0.5)	0 (0)	3 (1.4)	
*gyrB*	0 (0)	0 (0)	1 (0.5)	
*23S rRNA*	0 (0)	1 (0.5)	0	

Complete, moderate, and lack of BI decline criteria were calculated by evaluating initial BI and BI at 1-year post-RFT. MB-multibacillary, PB-paucibacillary. *Values represented in mean (range), BI-bacillary index, RFT-released from treatment, N (%)-number (percentage), AMR-anti microbial resistance.

### Molecular AMR analysis

*WHO-recommended DRDRs*: AMR strains of *M. leprae* were identified only in Guizhou, Hunan, and Yunnan, and none of them was identified in Sichuan (Figure 2 and Table 3). Amongst the 290 cases, 11 (3.7%) strains showed drug resistance, and the remaining (96.3%) were sensitive to all the drugs. Drug resistance analysis of the 290 cases revealed eight single drug-resistant (seven, 2.4% dapsone and one, 0.34% ofloxacin) and two double drug-resistant (one, 0.34% each to dapsone and ofloxacin, rifampicin and ofloxacin) strains of *M. leprae*. Another double drug resistance to ofloxacin and clarithromycin was observed in one strain (0.34%). No correlation of drug resistance proportion was identified in male vs. female (9/200 vs. 2/90; *p* = 0.512), nerve vs. no nerve involvement (10/220 vs. 1/70; *p* = 0.470), deformity vs. no deformity (3/103 vs. 8/187; *p* = 0.751), and reaction vs. non-reaction (1/40 vs. 10 /250; *p* = 1.000) cases. None of the mutated strains was harboured in PB. The AMR strains were detected in only LL and BL cases (5/72 vs. 6/136; *p* = 0.518). The frequency of drug resistance in relapse cases was higher compared to that of new cases (3/20 vs. 8/270; *p* = 0.032). Interestingly, strains from new cases showed single drug resistance to dapsone or ofloxacin, while relapse cases exhibited double drug resistance to dapsone and ofloxacin, rifampicin and ofloxacin, and ofloxacin and clarithromycin (Table 3).

**Table 3.** Clinical characteristics and other relevant information of all AMR cases of leprosy.

Province	Case type	Sex	Age	WHO	Previous treatment history	R-J	Nerve	Deformity	Reaction	BI at diagnosis	BI at RFT	BI at1 year post RFT	Mutation results							
													Nth (DNA repair)	folP1 (Dapsone)	rpoA (Rifampicin)	rpoB (Rifampicin)	rpoC (Rifampicin)	gyrA (Ofloxacin)	gyrB (Ofloxacin)	23s rRNA (Clarithromycin)
Hunan	New	M	47	MB	–	BL	Y	Y	N	3.4	2.8	2.4	–	55; CCC-TCC (Pro-Ser)	–	–	–	–	–	–
Hunan	New	M	37	MB	–	BL	Y	Y	Y	3.2	2.5	1.5	–	53; ACC-AGA (Thr -Arg)	–	–	–	–	–	–
Hunan	New	M	30	MB	–	BL	Y	N	N	5.2	4	3	–	53; ACC-ATC (Thr-Ile)	–	–	–	–	–	–
Guizhou	New	M	38	MB	–	LL	N	N	N	5.4	2.8	2.2	–	55; CCC-TCC (Pro-Ser)	–	–	–	–	–	–
Guizhou	New	M	41	MB	–	BL	Y	N	N	1.5	3.2	3.2	–	–	–	–	–	91; GCA-GTA (Ala-Val)	–	–
Guizhou	New	M	18	MB	–	LL	Y	N	N	3.7	UT	UT	–	55; CCC-TCC (Pro-Ser)	–	–	–	–	–	–
Guizhou	Relapse	F	70	MB	MDT	LL	Y	N	N	4.2	UT	UT	–	55; CCC-CGC (Pro-Arg)	–	–	–	91; GCA-GTA (Ala-Val)	–	–
Guizhou	Relapse	M	61	MB	MDT	BL	Y	N	N	2.2	1.0	0.7	–	–	–	410; GAT-TAT (Asp-Tyr)	–	91; GCA-GTA (Ala-Val)	–	–
Yunnan	New	M	85	MB	–	BL	Y	N	N	3.2	0	0	–	55; CCC-TCC (Pro-Ser)	–	–	–	–	–	–
Yunnan	Relapse	M	45	MB	DDS mono	LL	Y	Y	N	4.0	2.0	2.2	–	53; ACC-ATC (Thr-Ile)	–	–	–	–	–	–
Yunnan	New	F	25	MB	–	LL	Y	N	N	3.5	UT	UT	–	–	–	–	–	91; GCA-GTA (Ala-Val)	–	A2143C
Guizhou	New	M	41	MB	–	BT	Y	N	Y	1.5	0	0	–	–	–	–	698; AAC- ACC (Asp–Thr)	–	–	
Yunnan	New	M	45	MB	–	BL	N	N	N	3.4	UT	UT	–	–	–	–	–	362; GGA- GAT (Gly–Asp)	–	
Yunnan	New	M	35	MB	–	BL	Y	N	Y	3	3	3	–	–	–	–	–	362; GGA- GAT (Gly-Asp)	–	
Yunnan	Relapse	M	78	MB	MDT	BL	Y	N	N	3.4	3.6	3.6	–	–	–	–	–	362; GGA- GAT (Gly–Asp)	–	
Yunnan	New	M	79	MB	–	BT	Y	N	Y	0.2	0	0	–	–	–	–	–	362; GGA- GAT (Gly–Asp)	–	
Yunnan	New	F	26	MB	–	BL	Y	N	N	3.6	3.6	3.6	–	–	–	–	–	–	214; GTG- GGG (Val– Gly)	
Hunan	Relapse	M	72	MB	MDT	LL	Y	Y	N	4.2	3.2	3.2	–	–	–	–	–	–	–	A2142C

No shaded, fully shaded, and light-shaded indicates mutations in WHO-recommended, both WHO and extended, and extended DRDRs genes respectively, M-male, F-female, Y-yes, N-no, UT-under treatment.

*Extended DRDRs*: Among 290 cases, no mutations were identified in *nth*, *rpoA*, and *rpoB*. The four single mutations in *gyrA* were detected in different strains (1.37%), whereas each of the *rpoC*, *gyrB*, and *23S rRNA* mutations were detected in one strain each (0.34%) (Table 3).

The screening of WHO-recommended and extended DRDRs revealed 18 mutated strains among 290 cases of leprosy. Similar to WHO-recommended DRDRs, no correlation of drug resistance proportion was observed in male vs. female (15/200 vs. 3/90; *p* = 0.200), nerve vs. no nerve involvement (16/220 vs. 2/70; *p* = 0.258), deformity vs. no deformity (4/103 vs. 14/187; *p* = 0.310), and reaction vs. non-reaction (4/40 vs. 14/250; *p* = 0.288) cases of leprosy. No mutations existed in PB, whereas all mutations were assigned to MB (18/270) cases. The TT and BB cases did not have mutations, whereas LL (6/72) followed by BL (10/136) and BT (2/53) harboured mutations (*p* = 0.583). The mutation frequency was significantly higher in relapse cases than that in new cases (5/20 vs. 13/270; *p* = 0.004) (Table 3)**.**

### Relationship between BI and AMR

Analysis of the relationship between the BI and AMR mutations in WHO-recommended DRDRs revealed one *folP1* mutated strain amongst complete BI decline cases, five strains of *folP1* mutations and one strain with double mutation in *rpoB* and *gyrA* amongst moderate BI decline cases, and another *gyrA* mutated strain amongst cases of lack of BI decline (*p* = 0.251) (Tables 2 and 3)**.** The mutated strain analysis of cases under treatment is shown in Supplementary Table 6.

The relationship between the BI and AMR mutations in DRDRs (WHO and extended) identified mutations in three out of 81 cases (*folP1*, *rpoC*, and *gyrA* mutated strain in each) with complete BI decline, seven out of 103 cases (five *folP1*, one *23S rRNA*, and one both *rpoB* and *gyrA*) with moderate BI decline, and four out of 34 cases (three *gyrA* and one *gyrB*) with lack of BI decline (*p* = 0.268) (Tables 2 and 3).

### The emergence of novel mutations

In addition to previously described mutations, one strain presented mutation Asp698Thr in *rpoC*, conferring rifampicin resistance. Gly362Asp in *gyrA* and Val214Gly in *gyrB* were novel quinolone resistance mutations identified in four and one strains of *M. leprae* respectively. Other mutations at A2142C and A2143C in *23S rRNA* conferring clarithromycin resistance were identified in one strain each. We further retrieved the mutations in previously treated cases and identified the emergence of Thr53Ile mutation in *folP1* in one patient previously treated with DDS monotherapy. Mutation analysis of other previous cases treated with MDT drugs and newly diagnosed cases with no previous history of MDT treatment revealed acquisition of different mutations, confirming the emergence of drug resistance of leprosy (Table 3).

### Geographical and genetic clustering analysis

Guizhou, Sichuan, and Yunnan have common geographical borders, whereas Hunan shares border only with Guizhou (Figure 2). A total of 18 strains of *M. leprae* with mutations were distributed across three provinces in 15 counties geographically well separated. The Qixingguan, Huayuan, and Guangnan counties had two mutated strains each identified in different villages and families. Further investigations revealed no evidence of the migration of any of the cases infected with these 18 strains at the village, county, and province levels.

**Figure 3.**
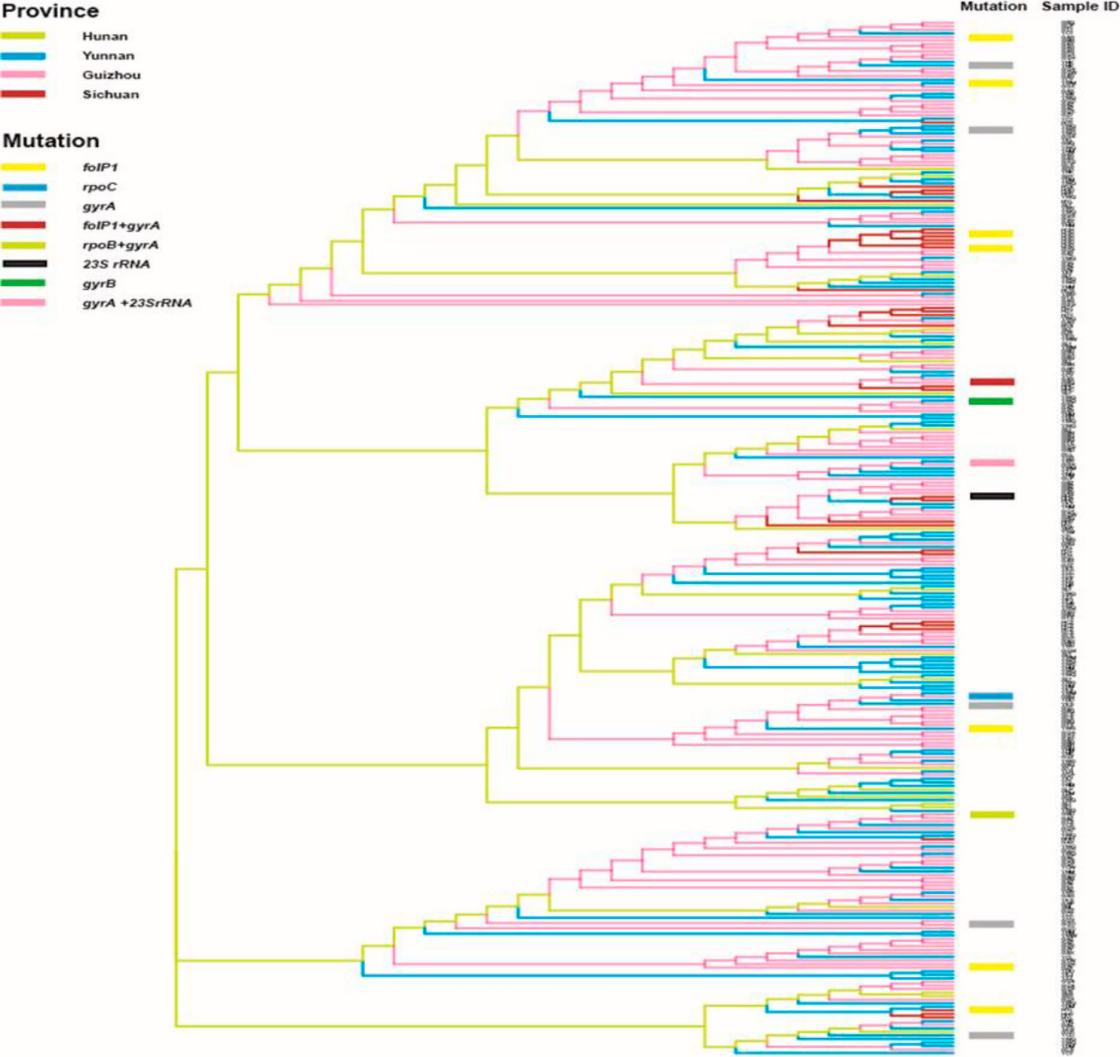
Phylogeny of wild-type and mutated strains from all four provinces. Genetic clustering pattern based on variable number tandem repeats for all wild-type and mutated strains (290 strains) of the counties of studied provinces. All branches are highlighted with four colours indicate four provinces. Mutated strains are represented with coloured rectangle columns. Each sample information is represented with three letters, where the 1st, 2nd, and 3rd letter specifies province, city, and county respectively.

**Figure 4.**
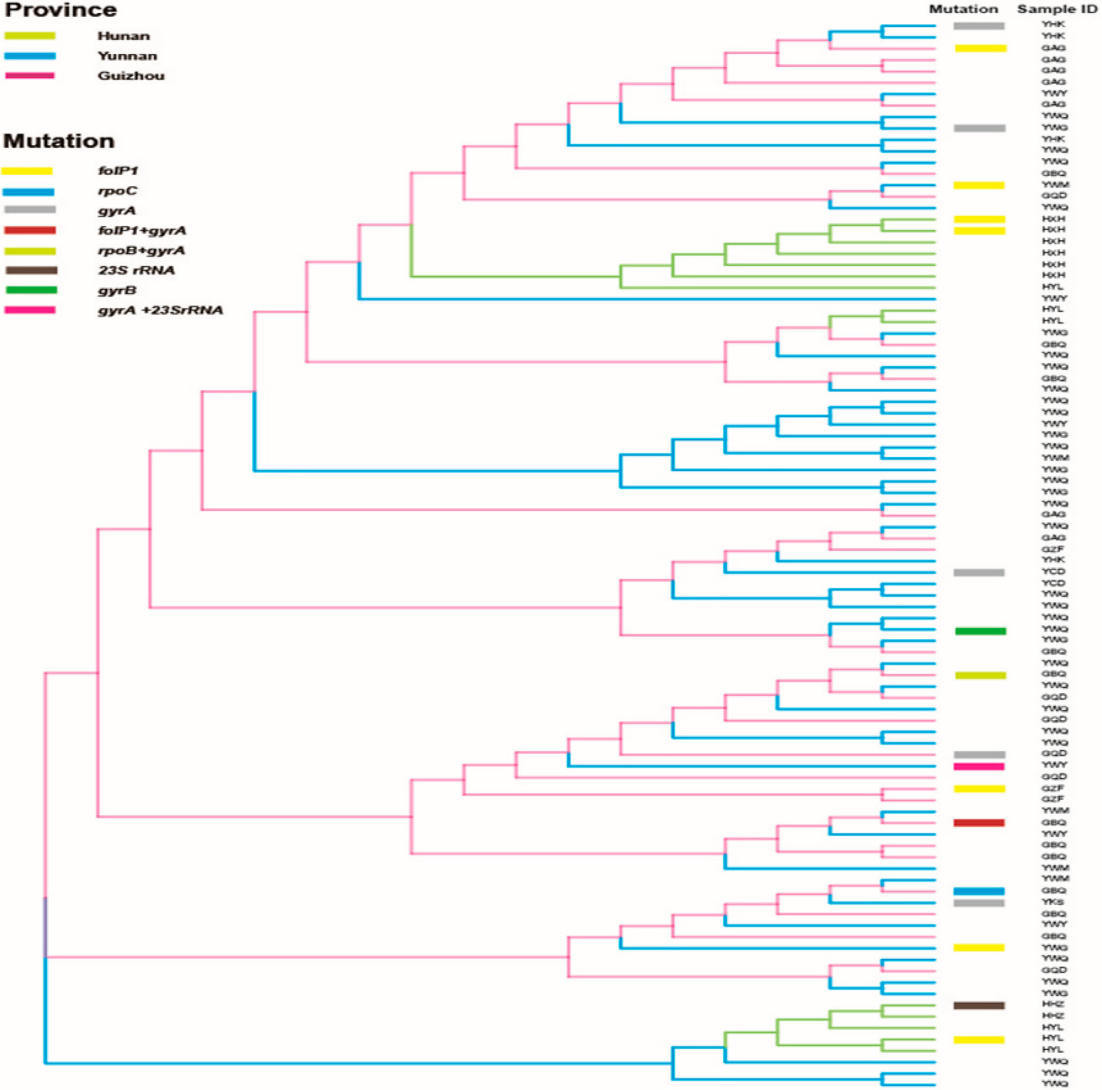
Phylogeny of wild-type and mutated strains from counties where drug resistance strains were detected. In counties where DR strains were detected, a phylogenetic relationship to local wild-type strains (94 strains) was examined based on their variable number tandem repeat genotypes. All branches are highlighted with four colours designate four provinces. Mutated strains are represented with coloured rectangle columns. Each sample information is represented with three letters, where the 1st, 2nd, and 3rd letter specifies province, city, and county respectively.

The VNTR genetic distances of *M. leprae* revealed that most of the mutated strains had unique genotypes and were also genetically well separated from wild-type strains (Figure 3). Genotype cluster analysis of wild-type and mutated strains of *M. leprae* at county level was performed using strains from patients residing in the counties where mutated strains were detected. We observed that the mutated strains had no clear cluster pattern, indicating genetic diversity even in the same county (Figure 4). Interestingly, one clear cluster was observed with wild-type (*n* = 5) and mutated strains (*n* = 2) in the Huayuan county of Hunan; however, mutated strains of this cluster originated from a different village, and no migration information of the patients was observed (Figure 4).

## Discussion

WHO declares that MDT treatment is effectively controlling leprosy incidence and contributing to the elimination of the leprosy burden in several countries, including China. The emergence of drug resistance in leprosy is a major concern for the implementation of disease intervention programmes. As very limited studies described the prevalence of gene mutations associated with AMR, the present study highlighted the characterization of the emergence of drug resistance by analysing DRDRs and some extended potential DRDR genes of *M. leprae* in China.

In newly diagnosed cases of leprosy, the high frequency of AMR to dapsone (six, 2.2%) in WHO-recommended DRDRs was identified in the present study*,* which was less than that in India [23], Korea [24], Myanmar, Indonesia, Philippines [25], Vietnam [26], and Brazil [3] but higher than that in Colombia [12] and China [27]. In this study, no mutation associated with rifampicin resistance was confirmed, whereas rifampicin resistance cases were confirmed in India [23], Brazil [28], China [27], Myanmar, Indonesia, and Philippines [25]. We reported that the AMR frequency for ofloxacin (two, 0.7%) was less in the current study leprosy population than that of Colombia [12], India [3], and China [27] leprosy population.

In relapse cases, the AMR to dapsone (one, 5%) and double mutation in both dapsone and ofloxacin (one, 5%) and rifampicin and ofloxacin (one, 5%) were less frequent than those found in Brazil [29] and Korea [24] but higher than those in India [23] and Colombia [12]. Other studies from Myanmar, Indonesia, Philippines [25], Vietnam [26], and South America [30] reported higher dapsone AMR cases than those observed in the present study. A previous study from China reported higher ofloxacin resistance than that described in the present study [27].

There was no significant correlation in AMR mutations in *M. leprae* in male vs. female, nerve vs. no nerve involvement, deformity vs. no deformity, and reaction vs. non-reaction. However, higher AMR mutations were recorded in male, nerve involvement, no deformity, and reaction cases of leprosy, in agreement with the findings of the other studies [12,16,28,29]. Similar to studies reported previously, no mutations in *M. leprae* were identified in PB, whereas all mutations were assigned to MB cases of leprosy [12,16]. In accordance with other studies, the TT and BB cases did not exhibit any mutations, whereas higher mutation frequency was identified in LL cases of leprosy [16,28,29]. The reported AMR mutations in *M. leprae* in different clinical forms of leprosy suggest that the situation needs to be carefully monitored with an effective surveillance system.

Mutations in extended DRDRs genes of *M. leprae*, including *rpoA*, *rpoB*, and *rpoC* were observed in *M. tuberculosis* [7], whereas only *rpoB* and *rpoC* mutations were found in *M. leprae* [8]*.* We identified only one *rpoC* mutation in *M. leprae.* Mutations at 362 of *gyrA* and 214 of *gyrB* conferring fluoroquinolone AMR were the first identified mutations in the Chinese leprosy population*.* We also reported the *23S rRNA* mutation conferring clarithromycin resistance in Chinese leprosy patients for the first time. The role of these mutations still requires further study to determine their phenotypic outcomes in *M. leprae*.

Considering well established national policies and socioeconomic factors, reduced transmission of both AMR and non-AMR cases of leprosy have been observed in China. Though dapsone monotherapy has been used in the pre-MDT era across China [31], the irregular use of this drug resulted in high dapsone resistance. It was believed that these resistant strains are continuously transmitted in the community [32]. Fortunately, rifampicin drug resistance is very low in the current study population. Patients administered with rifampicin for the treatment of other diseases and irregular MDT may explain the rifampicin resistance in China [33]. Fluoroquinolones are promising and widely used antibiotics introduced into routine clinical practice to treat infectious diseases in China. Given the easy availability and inappropriate use of these drugs, AMR to fluoroquinolones has emerged in China [34]. Clarithromycin is another antibiotic used to treat various bacterial infections [35,36], and it is used as an alternative or combinational drug that can result in clarithromycin resistance of leprosy [37].

Generally, patients who remain BI zero or become negative do not develop any positive indication for AMR. However, in the current study, we observed *folP1*, *rpoC*, and *gyrA* mutated strains in three different cases with BI zero. These findings revealed that BI negative cases might also have active bacteria showing drug resistance. Hence, careful monitoring of BI negative cases of leprosy is necessary for drug resistance surveillance. Generally, a lack of BI decline is an indicator of drug resistance, but the present study demonstrated that drug resistance might not be the main factor for lack of BI decline. BI decline is gradual and can take several years to become negative. Therefore, examining bacterial viability by mouse footpad culture, morphological index, and molecular tests may be necessary for establishing the lack of BI decline in leprosy.

Genotype analysis can be used to trace the transmission of *M. leprae* strains [38]. Isolates forming clear clusters of a certain strain type commonly demonstrate the recent and ongoing transmission of leprosy [38]. Most of the strains of *M. leprae* from Guizhou, Yunnan, and Hunan exhibited more clear clusters than those from Sichuan at the province level (Figure 3), implying a recent transmission and being the major drive for maintaining leprosy at the province level in China. The genotyping cluster analysis of both wild-type and mutated strains of *M. leprae* in leprosy cases obtained from the counties where mutated strains were detected revealed that the mutated strains had less cluster pattern with wild-type strains at the county level, indicating the existence of genotypically diverged AMR strains of *M. leprae* even in the same county-level (Figure 4). Hence, genotypically diverged strains with less clustering patterns denote no active transmission of AMR strains of leprosy at the county level. Interestingly, one clear cluster was observed with wild-type (*n* = 5) and mutated strains (*n* = 2) from Huayuan county of Hunan. These cases were analysed for geographical landscape and migration history at the village and county levels. The patients carrying the mutated strains of this cluster were from a different village, and no migration information of the patients amongst these villages was observed. Remaining patients carrying wild-type strains were also evaluated to the mutated cases for their contact and migration information, and there was no knowledge of contact and migration information. Thus, these cluster cases might not have the same infectious source and are supposed to have acquired the infection at an earlier stage or by a divergent transmission line at the village and county levels where many generations of leprosy cases resided. China is operating very effective leprosy control programmes at the county level to eliminate leprosy, led to decreased transmission and thus further lined up the less cluster formation of strains and AMR strains of leprosy at county levels. Thus, molecular epidemiological analysis revealed low ongoing transmission of AMR strains of leprosy at the village and county levels in China.

Our AMR surveillance study provides additional information to the leprosy control programmes in China. The emergence of drug resistance in newly diagnosed cases with no previous treatment history of any drugs confirms the ongoing transmission of primary AMR strains of leprosy in China. Given the high mutation frequency in relapse cases, the surveillance of relapse cases should be highly prioritized. Based on our results, we recommend systemic screening of AMR associated mutations when prescribing either WHO-recommended MDT or alternative drug treatment for leprosy. While considering alternative drugs to dapsone and rifampicin in MDT, clarithromycin may be a promising choice as it is associated with low AMR mutation frequency of leprosy in China. We also reported that AMR mutation might not be the main factor for the lack of BI decline in leprosy. Thus, the mechanism underlying the phenomenon of lack of BI decline in leprosy should be investigated. Furthermore, molecular epidemiological analysis revealed low ongoing transmission of mutated strains at the county and village levels in China. However, interventions need to be implemented for tracing close contact of leprosy to prevent further transmission of mutated strains.

## Ethics approval and consent to participate

This study was approved by the institutional ethical committee and informed consent was obtained from all participants.

## Supplementary Material

Supplemental Material
