# Supplementary material for: Molecular surveillance of antimicrobial resistance and transmission pattern of Mycobacterium leprae in Chinese leprosy patients
Source: Emerg Microbes Infect. 2019 Oct 17;8(1):1479–89. doi: 10.1080/22221751.2019.1677177 (PMC6818117; doi:10.1080/22221751.2019.1677177)

**Molecular surveillance of antimicrobial resistance and transmission pattern of *Mycobacterium leprae* in Chinese leprosy patients**

**S1 table.** Patients treated with ≥12 months of MDT and their BI status at end of the treatment

| **Country** | **Number of cases treated with ≥24 months MDT** | **BI range at the end of the treatment** | **First author** |
| --- | --- | --- | --- |
| China (current study) | 34 | 0.2-5.8 | Santosh Chokkakula |
| China | 5 | 0.3 to 2.0 | Shen J |
| Colombia | 59 | 0.1 to 2.0 | Guerrero MI |
| Colombia | 36 | Positive* | Beltrán-Alzate C |
| India | 9 | 0 to 3.8 | Job CK |
| India | 50 | 2 to 6 | Hasanoor Reja AH |
| India | 10 | 2 to 5.5 | Sekar B |
| India | 63 | Positive* | Lavania M |
| Mexico | 6 | Positive* | Matsuoka M |
| Nepal | 6 | Positive* | Sapkota BR |
| Philippines | 219 | 0 to 4.3 | Balagon MF |

MDT-multi drug therapy, BI- bacteriological index. *Cases reported with positive BI at end of the MDT, but BI range was not available.

**S2 table.** List primers for amplification of WHO recommended DRDRs genes

| **Name** | **Primer sequence** | **Product length(bp)** |
| --- | --- | --- |
| *folP1*F-Out | CAATTCGTTCTCAGATGGCGG | 343 |
| *folP1*R-Out | CATCAACACCCACGCAACAC |  |
| *folP1*F-In | CTTGATCCTGACGATGCTGT | 254 |
| *folP1*R-In | CCACCAGACACATCGTTGAC |  |
| *rpoB*F-Out | AGCGGATGACCACCCAGGA | 406 |
| *rpoB*R-Out | TCTTCCTCGTCAGCGGTCAA |  |
| *rpoB*F-In | GTCGAGGCGATCACGCCGCA | 279 |
| *rpoB*R-In | CGACAATGAACCGATCAGAC |  |
| *gyrA*F-Out | GCGCAGCTATATTGATTACGCG | 387 |
| *gyrA*R-Out | GCTCCAGTAACGATATCACC |  |
| *gyrA*F-In | AAGTCCGCGATGGTCTCAAA | 263 |
| *gyrA*R-In | ACAATAACGCATCGCTGCC |  |

F- forward primer, R-reverse primer, Out- outer primer used for the 1^st^ PCR, In- inner primer used for the 2^nd^ PCR.

**S3 table.** List primers used for amplification of extended DRDRs genes

| **Gene name** | **Primer sequence** | **Product length(bp)** |
| --- | --- | --- |
| *nth*F | AAAGTAATGCCCCTCACGCC | 762 |
| *nth*R | AAGGTCTTCATTGCGGTCCC |  |
| *rpoA*F | TCAAATAGTGGGTGCCGAGAA | 1044 |
| *rpoA*R- | TTGGGCATTGCTGACACTCC |  |
| *rpoB*F | AGGTTCCGGGGCTACTTGAT | 2195 |
| *rpoB*R | CACCTCATCGGAGACGTTGG |  |
| *rpoC*F | ACCTGCTTGGTAAGCGTGTT | 920 |
| *rpoC*R | GTAGAAACCGGCGTCCTTGA |  |
| *gyrA*F1 | GGTACATCGTCGGGTCTTGT | 1040 |
| *gyrA*R1 | ACACGACTCCGAATTCAAGCA |  |
| *gyrA*F2 | TTCTATCGCTGAGCAAGTCCG | 1356 |
| *gyrA*R2 | ATCGAGGTGCGTTTAGCGTA |  |
| *gyrBF* | GCCGAATTCAAGAAGTGGCTT | 969 |
| *gyrBR* | TTCCAGTACCCAATGCCGTA |  |
| *23S rRNA*F1 | TAAAAACACAGGTCCGTGCG | 180 |
| *23S rRNA*R1 | CGAGCATCTTTACTCGTAGTGC |  |
| *23S rRNA*F2 | GGCGAGTGTAAATGCACAAGG | 182 |
| *23S rRNA*R2 | CGGCGGATAGAGACCGAA |  |

*gyrA1* and *gyrA2* primer sets were used to cover the entire *gyrA* gene *23SrRNA1* and *23SrRNA2* primer sets amplify non-contiguous DRDRs of *23SrRNA* gene.

**S4 table.** Reaction mixture for amplification WHO recommended and extended DRDRs genes

| **Ingredients** | **WHO**  **recommended**  **1^st^ PCR** | **WHO recommended**  **2^nd^ PCR (nested PCR)** | **Extended** |
| --- | --- | --- | --- |
| Master mix | 12μL | 12μL | 12μL |
| Nuclease free H_2_0 | 4μL | 6 μL | 4μL |
| Forward primer | 1μL | 1μL | 1μL |
| Reverse primer | 1μL | 1μL | 1μL |
| Genomic DNA | 2 μL | 0.5 μL | 2 μL |
| Total volume | 20 μL | 20 μL | 20 μL |

**S5 table.** Reaction condition for amplification of WHO recommended DRDRs genes

| **Conditions** | ***folP1*** | ***rpoB*** | ***gyrA*** |
| --- | --- | --- | --- |
| Initial denaturation | 95°C for 10 min | 95°C for 10 min | 95°C for 7 min |
| Denaturation | 95°C for 30 s | 95°C for 30 s | 94°C for 30 s |
| Annealing | 58°C for 30 s | 60°C for 30 s | 56°C for 40 s |
| Elongation | 72°C for 60 s | 72°C for 60 s | 72°C for 60 s |
| Final elongation | 72°C for 10 min | 72°C for 10 min | 72°C for 10 min |
| hold | 4°C | 4°C | 4°C |
| Cycles | 39 | 35 | 39 |

The conditions are same for both 1^st^ and 2^nd^ PCR.

**S6 table.** Reaction condition for amplification of extended DRDRs genes

| **Conditions** | ***nth*** | ***rpoA*** | ***rpoB, rpoC,gyrA, gyrB and 23S rRNA*** |
| --- | --- | --- | --- |
| Initial denaturation | 95°C for 5 min | 95°C for 5 min | 95°C for 5 min |
| Final denaturation | 95°C for 40 s | 95°C for 40 s | 94°C for 40 s |
| Annealing | 63°C for 30 s | 58°C for 30 s | 55°C for 40 s |
| Elongation | 72°C for 3 min | 72°C for 3 min | 72°C for 3 min |
| Elongation | 72°C for 10 min | 72°C for 10 min | 72°C for 10 min |
| hold | 4°C | 4°C | 4°C |
| Cycles | 35 | 35 | 35 |

**S6 table.** Clinical characteristics and drug resistance analysis of patients under treatment

| Clinical characteristics | Hunan  N (%) | Guizhou  N (%) | Yunnan  N (%) |
| --- | --- | --- | --- |
| Case type |  |  |  |
| New | 1 (1.4) | 37 (51.4) | 28 (38.9) |
| Relapse | 0 (0) | 4 (5.5) | 2 (2.8) |
| Gender |  |  |  |
| Male | 0 (0) | 27 (37.5) | 18 (25) |
| Female | 1 (1.4) | 14 (19.4) | 12 (16.7) |
| Age* | 72 | 40 | 45 |
| WHO classification |  |  |  |
| MB | 0 (0) | 40 (55.5) | 28 (38.9) |
| PB | 1 (1.4) | 1 (1.4) | 2 (2.8) |
| R J classification |  |  |  |
| TT | 1 (1.4) | 1 (1.4) | 0 (0) |
| BT | 00 | 6 (8.3) | 7 (9.7) |
| BB | 00 | 1 (1.4) | 1 (1.4) |
| BL | 00 | 17 (23.6) | 18 (25) |
| LL | 00 | 16 (22.2) | 4 (5.6) |
| BI, range(mean) | 0 (0) | 0-5.4 (3.13) | 0-5 (2.7) |
| Nerve involvement |  |  |  |
| Yes | 0 (0) | 33 (45.8) | 20 (27.8) |
| No | 1 (1.4) | 8 (11.1) | 10 (13.9) |
| Deformity |  |  |  |
| Yes | 0 (0) | 19 (26.4) | 11 (15.2) |
| No | 1 (1.4) | 22 (30.6) | 19 (26.4) |
| Reaction |  |  |  |
| Yes | 0 (0) | 1 (1.4) | 6 (8.3) |
| No | 1 (1.4) | 40 (55.6) | 24 (33.3) |
| 16sRNA PCR |  |  |  |
| Positive | 1 (1.4) | 41 (56.9) | 30 (41.7) |
| Negative | 0 | 0 | 0 |
| Drug resistance |  |  |  |
| *folP1* | 0 | 1 (1.4) | 0 |
| *gyrA* | 0 | 0 | 1(1.4) |
| *folP1* + *gyrA* | 0 | 1 (1.4) | 0 |
| *gyrA*+ *23S rRNA* | 0 | 0 | 1 (1.4) |

*The values represented in mean; N (%) values described number (percentage), MB- multibacillary, PB- paucibacillary, TT-tuberculoid tuberculoid leprosy, BT- borderline tuberculoid leprosy, BB- borderline borderline leprosy, BL- borderline lepromatous leprosy, LL- lepromatous leprosy.

**S1** **figure**. Comparison of BI distribution at 3-time points Initial, RFT and 1 year post RFT


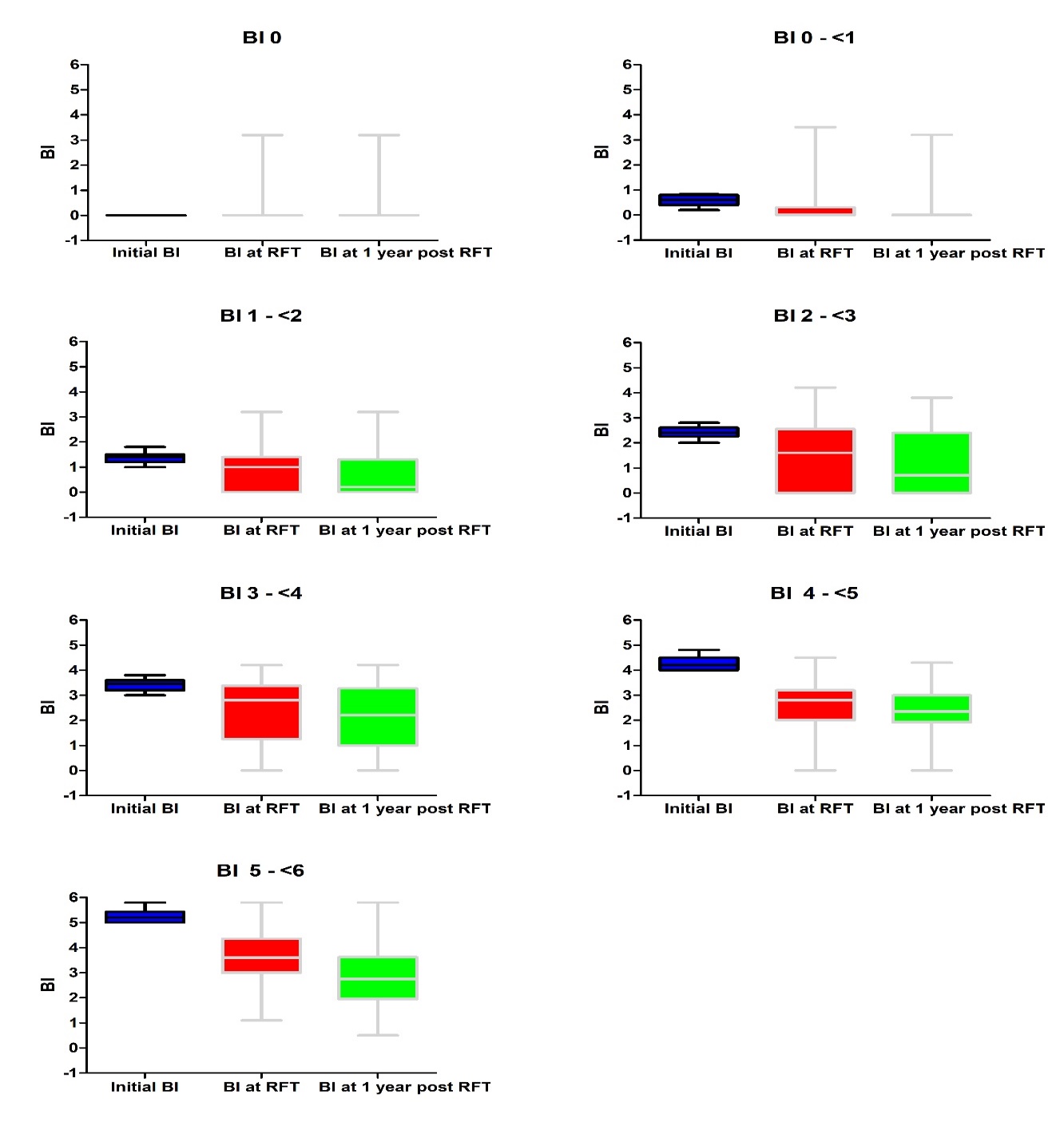


Different BI measures were reported at 3 time point at initial, RFT and 1 year post RFT. BI- bacteriological index, RFT- released from the treatment.


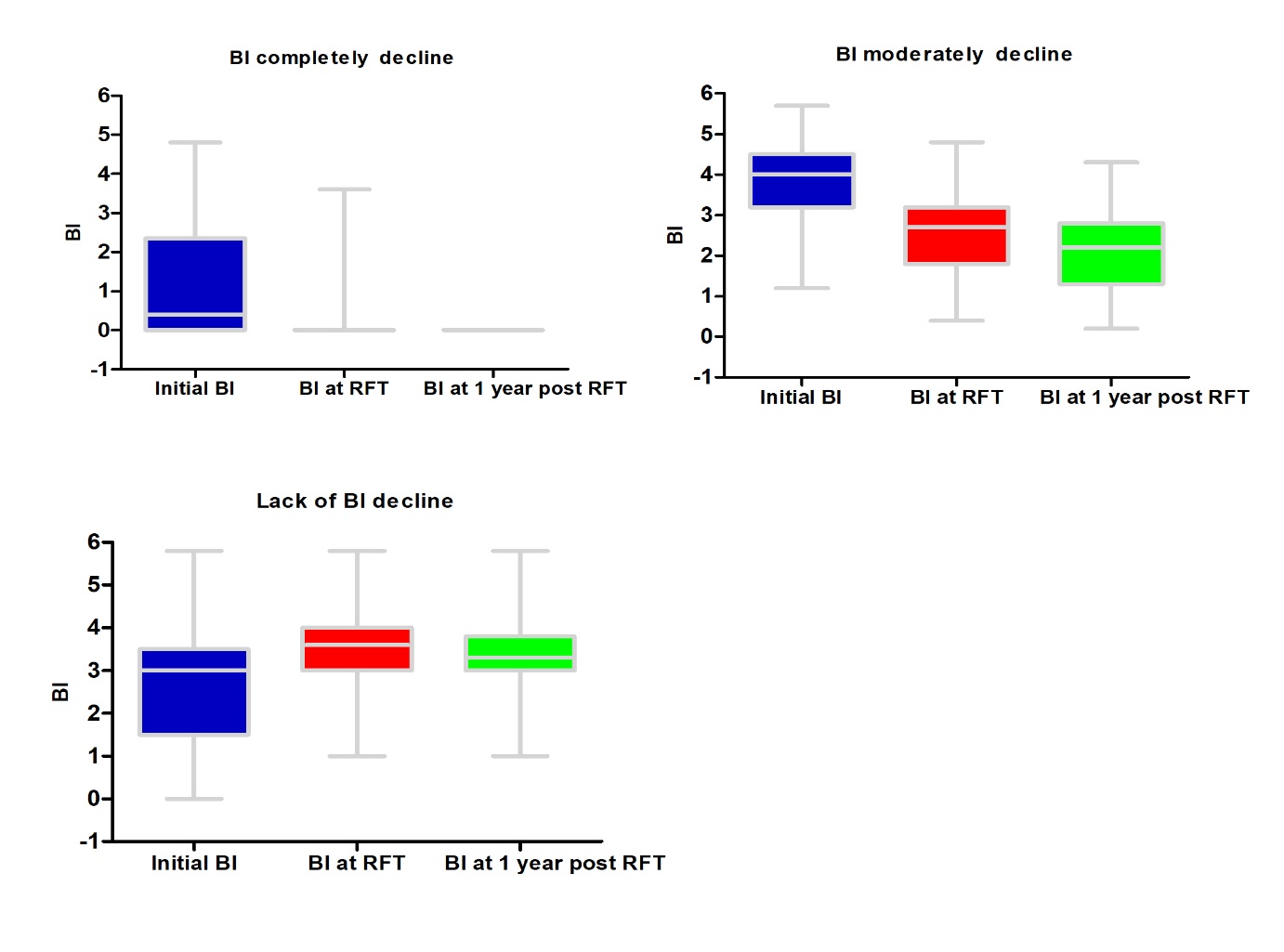
**S2 figure**. BI distribution in BI completely decline, BI moderately decline and BI no change cases at 3-time points Initial, RFT and 1 year post MDT

Three categories -BI completely decline, BI moderately decline and BI no decline were categorized according to BI measured at three-time points initial, RFT and 1 year post RFT. BI- bacteriological index, RFT- released from the treatment.

**S3 figure.** Chromatogram files of DRDR and extended DRDR gene sequences with different types of mutations.

53 *folP1* (ACC-AGA)

*53 folP1* (ACC-ATC)


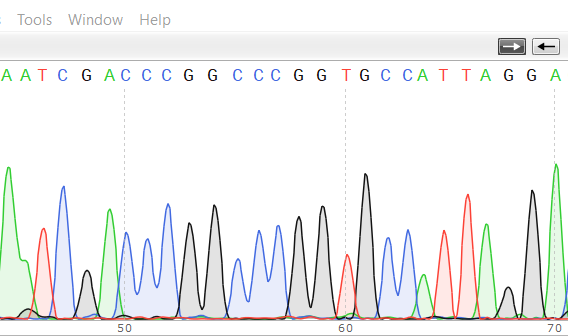

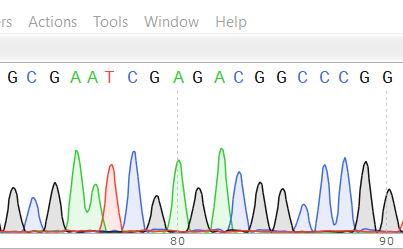

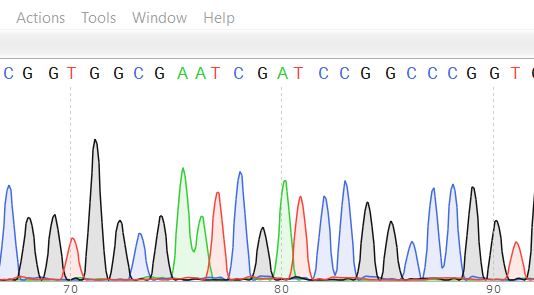

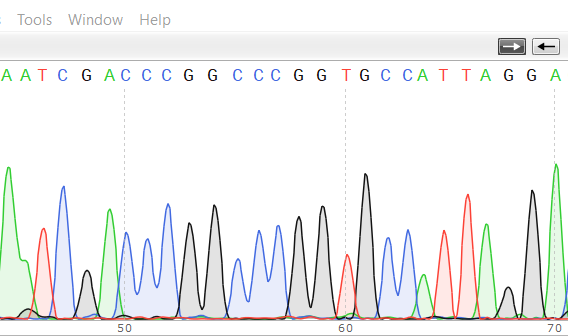


55 *folP1 (*CCC-TCC)

55 *folP1* (CCC-CGC)


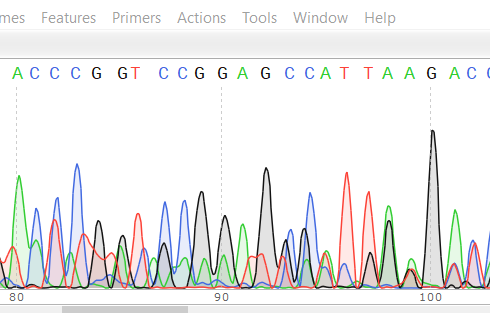

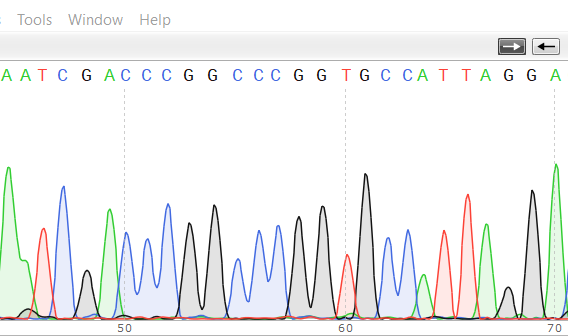

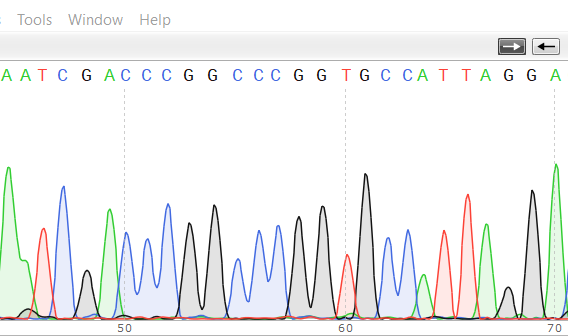

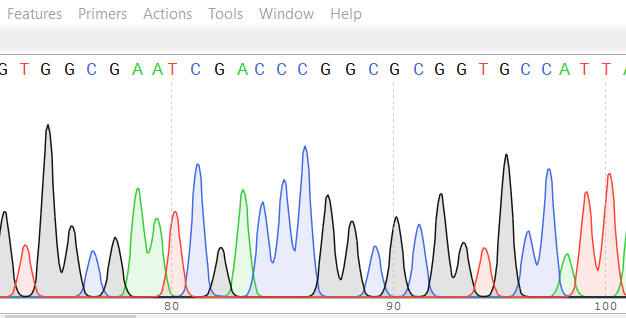


410 *rpoB (*GAT-TAT)

91 *gyrA (*GCA-GTA)


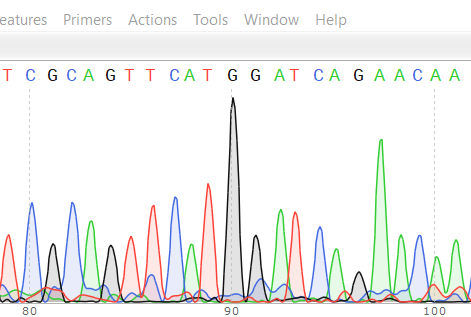

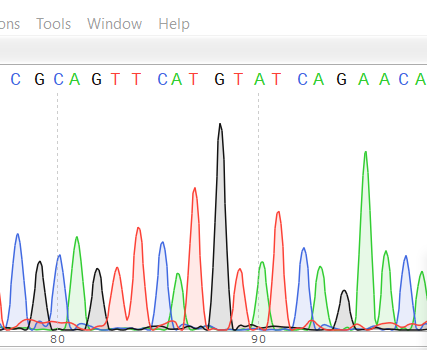

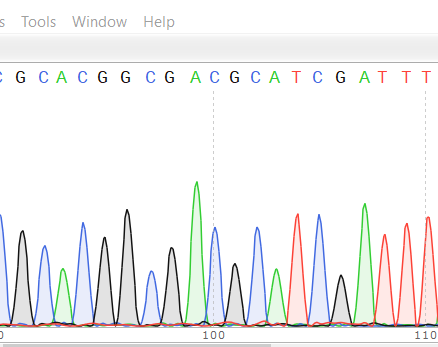

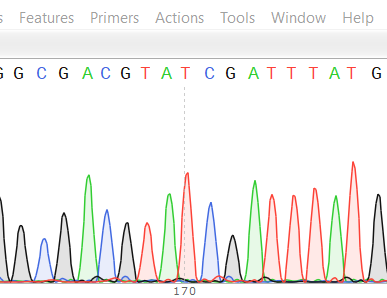


362 *gyrA (*GGA-GAT)

698 *rpoC* (AAC-ACC)


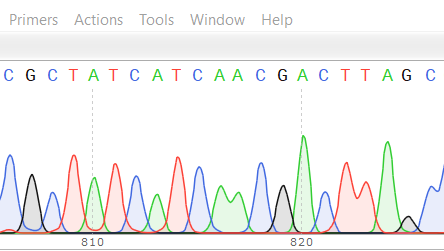

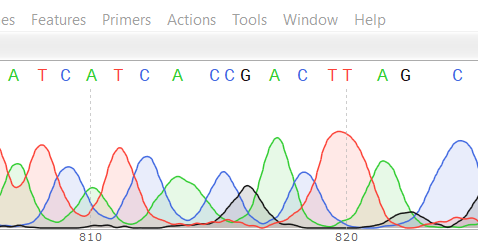

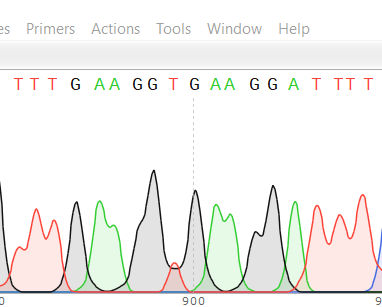

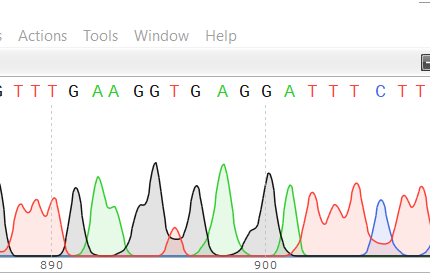


*214 gyrB* (GGA-GGG)

*23S rRNA (*A2142 C)


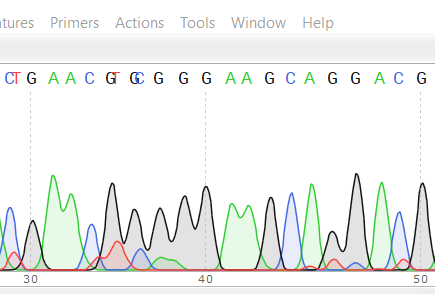

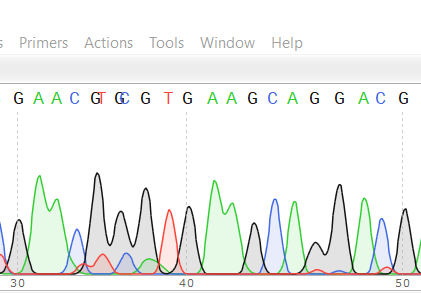

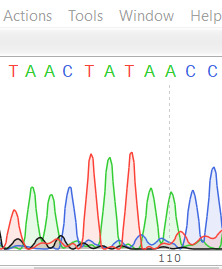

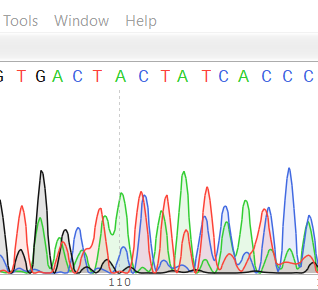


*23S rRNA (*A2143C)


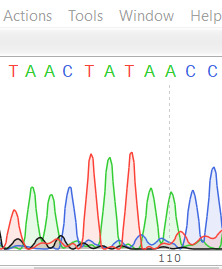

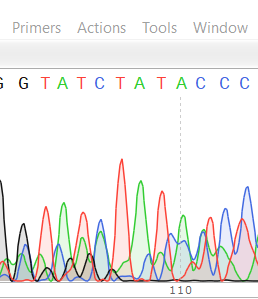

Supplement: Supplemental Material [file TEMI_A_1677177_SM0758.docx]
